# A randomized controlled trial evaluating the effectiveness of a self-management program for adolescents with a chronic condition: a study protocol

**DOI:** 10.1186/s13063-022-06740-9

**Published:** 2022-10-05

**Authors:** Jaunna Gauci, Jacqueline Bloomfield, Sharon Lawn, Susan Towns, Annabelle Hobbs, Katharine Steinbeck

**Affiliations:** 1 Department of Adolescent Medicine, The Children’s Hospital at Westmead, Locked Bag 4001, Westmead, Sydney, NSW 2145 Australia; 2 Discipline of Child & Adolescent Health, Sydney Medical School, The University of Sydney, Sydney, Australia; 3 Susan Wakil School of Nursing and Midwifery, Faculty of Medicine and Health, The University of Sydney, Sydney, NSW Australia; 4 College of Medicine and Public Health, Flinders University, Adelaide, SA Australia; 5 The Academic Department of Adolescent Medicine, The Children’s Hospital at Westmead, Sydney, Australia

**Keywords:** Adolescent, Chronic illness, Chronic condition self-management, Flinders Program, Therapy adherence, Concordance, Patient compliance, Randomized controlled trial

## Abstract

**Background:**

Self-management support is increasingly viewed as an integral part of chronic condition management in adolescence. It is well recognized that markers of chronic illness control deteriorate during adolescence. Due to the increasing prevalence of long-term chronic health conditions in childhood and improved survival rates of previously life-limiting conditions in children and adolescents, significant numbers of adolescents are having to manage their chronic condition effectively as they transition to adult health care. Therapy adherence has been identified as a major challenge for young people living with a chronic condition such as cystic fibrosis, diabetes, or asthma requiring long-term pharmacological therapy and/or lifestyle modifications. Most systematic reviews on self-management interventions address adult populations. Very few intervention studies are directed at adolescents with a chronic condition who are transitioning to adult health services. This protocol describes a prospective randomized controlled trial of a standardized self-management intervention program delivered to adolescents aged 15–18 years prior to their transfer to adult care. This study has been designed to provide evidence regarding self-management programs for adolescents and is the first study to use the Flinders Program with this important, under-researched age group.

**Methods:**

A randomized controlled trial is used to investigate the effectiveness of a modified adolescent-friendly version of an adult self-management program. This program is directed at improving self-management in an adolescent cohort 15–18 years of age with a chronic condition being treated in a specialist pediatric hospital. Participants will be randomized to either usual care or the modified Flinders Program plus usual care. Data collection will include measures of specific illness control, unscheduled hospital admissions, and questionnaires to record self-management competencies, quality of life, self-efficacy, and outcome measures specific to the chronic condition at baseline, 3 months, 6 months, and 12 months after delivery.

**Discussion:**

This study will provide a better understanding of the elements required for effective self-management programs in adolescents with a chronic condition and address some important knowledge gaps in current literature. The study will be carried out in collaboration with the Discipline of Behavioural Health at Flinders University, Adelaide, Australia, in order to inform the development of an adolescent version of the successful and validated Flinders Program™.

**Trial registration:**

Australian and New Zealand Clinical Trials Registry (ACTRN12621000390886). Registered on April 8, 2021.

**Supplementary Information:**

The online version contains supplementary material available at 10.1186/s13063-022-06740-9.

## Administrative information

Note: The numbers in curly brackets in this protocol refer to the SPIRIT checklist item numbers. The order of the items has been modified to group similar items (see http://www.equator-network.org/reporting-guidelines/spirit-2013-statement-defining-standard-protocol-items-for-clinical-trials/).

**Table:** 

Title {1}	A randomized controlled trial evaluating the effectiveness of a self-management program for adolescents with a chronic condition: a study protocol
Trial registration {2a and 2b}.	Australian and New Zealand Clinical Trials Registry, (ACTRN12621000390886). Registered on April 8, 2021. https://www.anzctr.org.au. Please refer to supplementary file 5, trial registration data for full details.
Protocol version {3}	April 22, 2021. Version: 3
Funding {4}	No external funding has been sourced.
Author details {5a}	**Jaunna Gauci,** Department of Adolescent Medicine, The Children’s Hospital at Westmead, Sydney, NSW, Australia, and Specialty of Child and Adolescent Health, The University of Sydney Medical School, Sydney, NSW, Australia T: + 61 2 9845 2446 e: jane.gauci@health.nsw.gov.au **A/Prof Jacqueline Bloomfield,** Susan Wakil School of Nursing and Midwifery, Faculty of Medicine and Health, The University of Sydney, Sydney, NSW, Australia. (e: jacqueline.bloomfield@sydney.edu.au **Prof Sharon Lawn,** College of Medicine and Public Health, Flinders University, Adelaide, South Australia. (e: sharon.lawn@flinders.edu.au) **A/Prof Susan Towns,** Department of Adolescent Medicine, The Children’s Hospital at Westmead, Sydney, NSW, Australia, and Specialty of Child and Adolescent Health, The University of Sydney Medical School, Sydney, NSW, Australia (e: susan.towns@health.nsw.gov.au **Dr Annabelle Hobbs,** Marie Bashir Clinical Research Fellow, The Academic Department of Adolescent Medicine, The Children’s Hospital at Westmead, Sydney, NSW, Australia. (e: annabelle.hobbs@qld.gov.au **Prof Katharine Steinbeck**, Specialty of Child and Adolescent Health, The University of Sydney Clinical School, The Children’s Hospital at Westmead, Sydney, NSW, Australia. e: Kate.steinbeck@health.nsw.gov.au
Name and contact information for the trial sponsor {5b}	Ms Jaunna Gauci Department of Adolescent Medicine, The Children’s Hospital at Westmead, Sydney Australia. Email: Jane.gauci@health.nsw.gov.au
Role of sponsor {5c}	N/A. There is not sponsor for this trial.

## Introduction

### Background and rationale {6a}

The concept of supporting self-management skills in those with a physical chronic condition has been recognized globally as an important health strategy in dealing with the growing burden of non-communicable disease (NCD), and the associated treatment costs of living with a chronic condition [1]. Advances in medical technology and medical treatments have led to improved survival rates across a range of chronic conditions [2]. As a result, the number of adolescents living with a chronic condition and disability has been steadily increasing in Australia and internationally [2, 3]. In 2009, there were nearly four million young people aged 12–24 years residing in Australia. This number is projected to increase to 4.8 million by 2038 [4]. According to the Australian Institute of Health and Welfare (AIHW) between 2007 and 2008, an estimated 60% of young people aged 12–24 years had a chronic condition. While a very broad definition was used, on current data at least 10% of these will have a longstanding chronic condition requiring significant self-management skills. Supporting the development of self-management skills has been well recognized in adults as an important health strategy for dealing with the burden of chronic illness. However, little is known about the effectiveness of self-management support programs for adolescents who are in the process of developing autonomous self-management skills [5].

Adolescence is characterized by rapid emotional, physical, psychosocial, and cognitive change as adolescents make the transition from childhood to young adulthood. In addition to the normal developmental challenges faced by all adolescents [2], young people with a chronic illness have the additional burden of managing their chronic condition along with its associated and often complex behaviors which the young person is expected to incorporate into their everyday life. These behaviors include regularly taking prescribed medications, monitoring symptoms, using medical devices, and lifestyle changes including nutrition and/or physical activity [6–10]. This therapy burden together with the often significant impact of a chronic illness on the young person’s physical, psychosocial, and even neurocognitive development often leads to non-adherence to treatment regimens [11–13]. Non-adherence is viewed as likely more challenging for young people than any other age group [7–9, 14–16]. The terms compliance, adherence, and concordance have been used interchangeably in the literature and are often used to describe self-management behaviors with notable differences between the terminology. Compliance is defined as “The extent to which the patient’s behavior matches the prescribers recommendations” [17]. However, its use is declining as it seems to imply lack of involvement by the patient. Adherence is defined as “the extent to which the patient’s behavior matches agreed recommendations from the prescriber” [13, 17, 18]. Concordance focuses on a collaborative agreement between the clinician and the young person about their treatment regimen, which supports components which are required for long-term management of their chronic condition with the emphasis on a collaborative partnership [18]. This aligns well with the principles of self-management.

The concept of self-management encompasses a combination of different strategies directed towards improving knowledge of their chronic condition together with the cognitive behavioral strategies which are required for long-term behavior change leading to improved health outcomes [19–22]. Studies in both adults and younger children with a chronic condition have demonstrated that combining self-management with medical treatment leads to improved health outcomes and better quality of life than prescriptive medical management alone [23].

Self-management has been defined as the actions undertaken by the individual on a daily basis to effectively manage their chronic condition and the associated impact of living with a chronic illness [24, 25]. The concept of promoting self-management skills involves much more than just providing didactic patient education alone. Rather, the young person is provided with skills that improve active management of their chronic condition. These include navigating the health care system, making independent decisions about their treatment, action planning, and developing self-efficacy and problems solving skills which can be applied to everyday situations [10, 19, 20, 26, 27]. Self-management support is the encouragement provided by health professionals, relatives, and significant others to increase the adolescent’s skills and confidence in managing their chronic condition [24, 25, 28–30]. Self-management is necessary for an effective transition to adult care, which is more than just a “transfer event” from pediatric to adult services. Rather it involves a process whereby adolescents develop the skills to carry out daily health care tasks independently in preparation for the transfer to adult services [31].

To date, there has been little attention directed towards the development of self-management in young people with a chronic illness, with the majority of research focusing on the adult population [7, 32, 33]. A study by Mammen and colleagues found that greater self-efficacy was demonstrated when self-management programs are precisely directed at the unique developmental needs of adolescents [34]. Adult models often overlook the developmental needs of young people which make it difficult to simply adapt these approaches to adolescents [10]. The overall goal of self-management programs is to provide a conceptual framework in which to deliver information and training and promote skills development, practical tools, and resources to improve self-efficacy and medical management, thereby improving the health outcomes of individuals living with a chronic condition [5, 10, 35]. Failure to effectively develop self-management skills in adolescence also has the potential to adversely influence future health trajectories in later life [15].

Managing a chronic condition and adapting behavior changes involves a collaborative approach between the young person and health care providers to enable more effective management of their chronic condition resulting in improved health outcomes [28]. It is this concept that drives the Flinders Program™ which is used in this study. From our experience, adolescents respond well to the Flinders Program™ in a clinical setting [36]. This study describes a prospective randomized controlled trial comparing a standardized self-management intervention program with usual care delivered to adolescents aged 15–18 years prior to their transfer to adult care and is the first study to use the Flinders Program™ with this important and under-researched age group.

### Objectives and hypothesis {7}

#### Primary research question

Does a modified adult chronic condition self-management program plus standard care improve therapy adherence in adolescents aged 15–18 years with a chronic condition compared to standard care only?

The *primary objective of this stud*y is to investigate the effectiveness via adherence of a modified adult chronic condition self-management support program in an adolescent population with a chronic condition within a tertiary pediatric hospital setting, compared to those receiving routine care services in the same setting using standardized markers of improvement in control of their chronic illness.

The *secondary objectives* include the use of routinely collected data on the number of unplanned or unscheduled health care or emergency department visits, together with questionnaire measures of quality of life, self-efficacy, time management, health-related distress, and feasibility and acceptability of the program to further refine its delivery under clinical conditions.

*Study hypothesis* to be tested is that a modified adult-focused, generic chronic condition self-management support program provided to adolescents with a chronic condition who are in the active phase of transition to adult services would:

1. Be more effective than standard usual care in achieving adherence to chronic condition management as measured by quantitative condition-specific validated markers of improvement in illness control as described in Table 1.
2. Be more effective than standard care in improving more global quality of life, self-efficacy, time management, and in reducing health-related distress as described in Table 2.

**Table 1:** Markers of illness control

Chronic condition	Measures of illness control	Definition of improvement^*^
Cystic fibrosis	▪ FEV1	FEV1% of predicted increased by ≥ 10%
Type 1 diabetes mellitus	▪ HbA1c	Absolute decrease in HbA1C by ≥ 1% (e.g., 9% reducing to 8%)
Inflammatory bowel disease	▪ Weight	Absolute increase in weight by ≥ 5%
Chronic kidney disease	▪ GFR	Absolute increase in GFR by ≥ 5%
Asthma	▪ FEV1	FEV1 % of predicted increased by ≥ 10%
Epilepsy	▪ Number of seizures	Based on individual and type of seizure as negotiated with treating Neurologist.

Abbreviations measures of illness control: *FEV1 (%)* forced expiratory volume in 1 s, *HbA1c* glycosylated hemoglobin (mmol/mol), *GFR* glomerular filtration rate (mL/min)

Weight (kilograms)

^*^Sustained improvement in measure of disease control over a 12-month period from baseline

**Table 2:** Schedule of enrolments, interventions, and assessments

Procedure												
**Measure**	**Baseline**		**3 months**		**6 months**		**9 months**		**12 months**		**18 months**	
	**I**	**C**	**I**	**C**	**I**	**C**	**I**	**C**	**I**	**C**	**I**	**C**
Informed consent	**X**	**X**										
Demographic data collection	**X**	**X**										
Measures of illness control	**X**	**X**	**X**		**X**	**X**		**X**	**X**	**X**		**X**
Unplanned hospital visits	**X**	**X**	**X**		**X**	**X**		**X**	**X**	**X**		**X**
Questionnaires	**X**	**X**	**X**			**X**		**X**	**X**			**X**
Partners in Health (PIH) Scale	**X**	**X**	**X**		**X**	**X**		**X**	**X**	**X**		**X**
Feasibility and Acceptability Questionnaire (FAQ)									**X**			**X**
Flinders Program delivery	**X**		**X**		**X**	**X**	**X**	**X**	**X**	**X**		**X**
Booster session					**X**					**X**		

***I*** Intervention (Flinders Program plus usual care), ***C*** Control (waitlist plus usual care)

### Trial design {8}

This is a pragmatic, two-site, two-arm, parallel, longitudinal randomized controlled trial, using both quantitative and qualitative research methods. Participants are allocated at a ratio 1:1 to the intervention or usual care using a computer-generated random list. An RCT design was chosen for this study due to its accepted applicability in assessing the effects of new healthcare interventions such as a self-management program [37, 38]. This study design has been shown to demonstrate sound methodological evidence [39], and allows for a comparison group at each time period and for analysis between each cluster [37, 40]. The study protocol received approval by The Sydney Children’s Hospitals Network Human Research Ethics Committee, Sydney, Australia. The study protocol has been developed based on the Standard Protocol Items: Recommendations for Interventional Trials (SPIRIT) guidelines Schulz and Grimes [41] (see Additional File 1). Figure 2 demonstrates the flowchart of the study design.

**Fig. 1:**
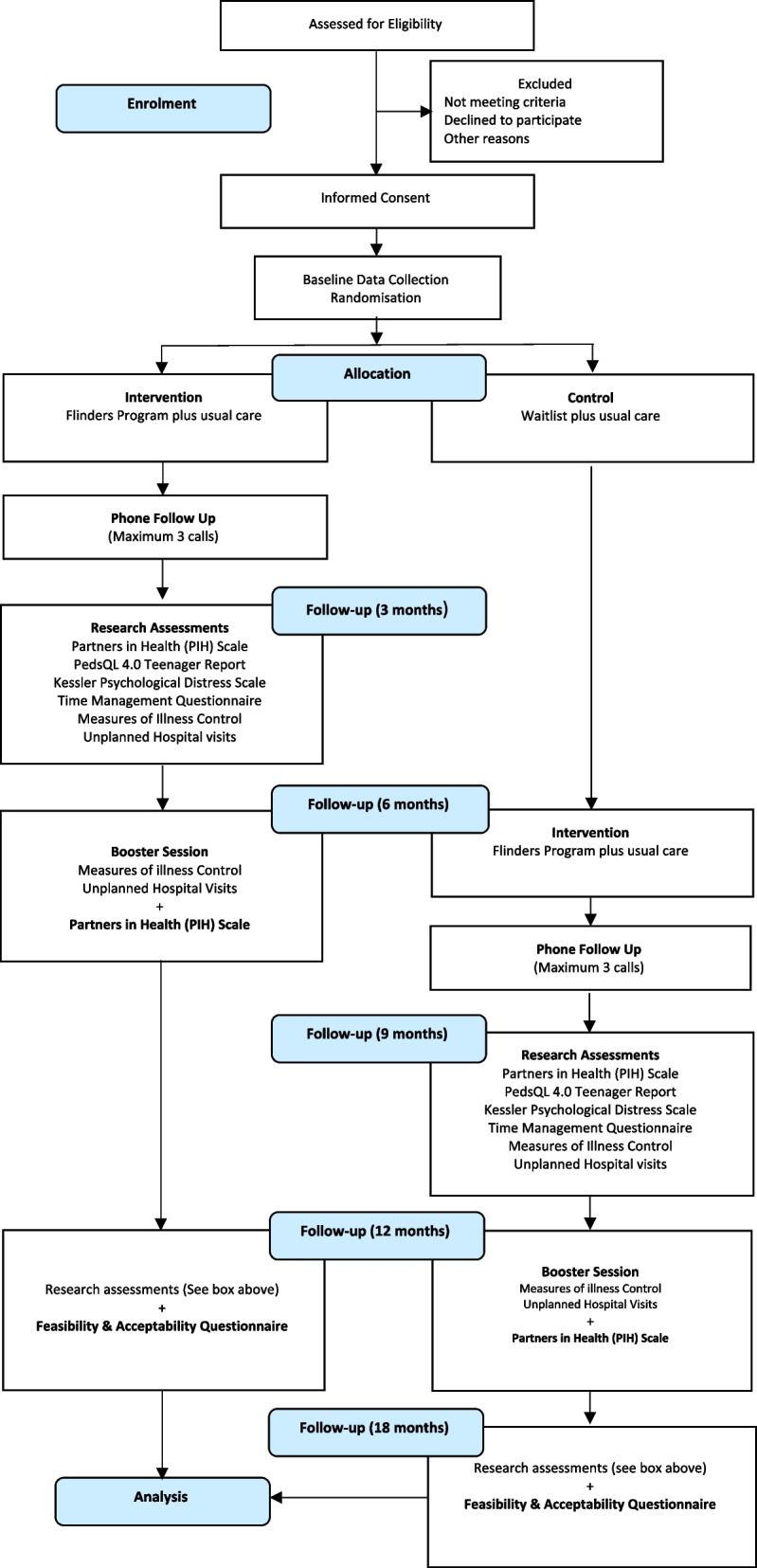
CONSORT (Consolidated Standards of Reporting Trials) flowchart for the self-management randomized controlled trial

This protocol describes an RCT to demonstrate the efficacy of the Flinders Program™ in adolescents. Following randomization, participants in the intervention group will receive a 12-month nurse-led modified version of the Flinders Program™ plus standard care, while participants in the waitlist control group will receive standard care for 6 months before crossing over to the intervention group until all participants are exposed to the program as demonstrated in the study design diagram in Fig. 1. Given that the data available suggests continuing deterioration without intervention is expected, we consider it ethically responsible to offer the intervention to both participant groups.

The study design will include a baseline collection period, followed by sequential, individual randomization to control or intervention. The intervention group and control group will be continually recruited and exposed to the intervention plus standard care and/or standard care with baseline and follow-up observations collected (see “Participant timeline {13}”) until target numbers are achieved (see sample size {14}). All participants will be asked to sign informed consent before the commencement of the study.

This is a pragmatic trial for the following reasons:

1. Our systematic review published in the Journal of Advanced Nursing (2021), provides evidence that self-management programs are effective for adolescents. We have been using the Flinders Program™ clinically for over 5 years with success in demonstrated effectiveness in self-management capacity. What is less well understood is how effective self-management programs remain over time, given the rapid bio-neuro-psychosocial development during adolescence. This study has the capacity to demonstrate duration of effect with 12-month follow-up planned.
2. Given the challenge of recruiting and retaining adolescents in health service research from the clinical experience of the research team and the clear clinical need to intervene [36], the combination of stepped intervention with assessment of duration of effect and guidance on booster sessions appears the more appropriate methodological option.

#### Study design diagram

Figure 1 shows the CONSORT (Consolidated Standards of Reporting Trials) flowchart for the self-management randomized controlled trial.

## Methods: participants, interventions and outcomes

### Study setting {9}

This study is being conducted within specialist clinics in two large tertiary pediatric university teaching hospital across the two campuses of the Sydney Children’s Hospital Network (SCHN) in Sydney, Australia. Participants will be recruited from The Children’s Hospital at Westmead and Sydney Children’s Hospital Randwick. The SCHN is the largest tertiary pediatric health care entity in Australia, providing world-class pediatric healthcare and research. In the 2019–2020 financial year, the SCHN provided care for over 153,000 children and young people, totalling over 50,000 hospital admissions and servicing different geographical areas of the state of New South Wales, Australia [42]. The population to be studied is adolescents aged 15–18 years of age with a chronic condition who have been identified by their treating team as requiring the development of self-management skills to effectively manage the challenges associated with their chronic illness.

### Eligibility criteria {10}

To be eligible for the trial, adolescents must meet the following criteria: (1) aged between 15 and 18 years; (2) have a chronic physical condition needing regular health care and requiring the development of self-management skills; (3) be identified by their treatment team as having unsatisfactory control of this condition. As we are an intervention service, at recruitment all our sample will have unsatisfactory control of their chronic condition based on the clinical judgment of their treating team and/or markers of illness control which is an inclusion criterion; (4) they must not have previously participated in a self-management program in the last 2 years. The rational for the choice of age criterion was based on the developmental stage of the adolescent. From middle adolescence (15–16 years) to late adolescence (17–19 years), young people are at a developmental stage where, from a neurocognitive perspective, they are better able to articulate and comprehend the information provided as their transition from pediatric to adult services is planned. From a developmental perspective, cognitively in early adolescence (12–14 years), it is challenging for an adolescent to conceptually understand and process the information provided in the Flinders Program™ as evident from our past clinical experience [36].

Eligible participants will be provided with a patient information sheet (PIS) and a consent form as identified by their primary treating team and then introduced to the research team. Parents/carers will also be provided with a parent information sheet describing the full details of the study. Ideally the consent will be provided face to face on the day. If the consent is not returned within 7 days, follow-up phone contact with the young person will be made by the research nurse to discuss consent.

The participant exclusion criteria include the following: (1) any adolescents who lack competence in English and are unable to understand and consent to participation in the study; (2) adolescents with an intellectual disability precluding independent self-management; (3) adolescents with any chronic condition with no routinely measurable quantitative condition-specific validated markers of illness control that can be used to identify clinically meaningful endpoints for example some metabolic disorders. The ineligible adolescents in this group will however be included in the self-management support clinic.

### Who will take informed consent? {26a}

The principal investigator retains the overall responsibility for ensuring that consent has been obtained from participants in the correct manner. The principal investigator may delegate this responsibility to a member of the research team with the appropriate training, experience, and knowledge to explain fully to potential research participants the implications of participating in the trial. Trial data will be collected from potential trial participants by a member of the research team via telehealth, in person or by phone or email if other methods are unobtainable. Following the confirmation of the participant eligibility and the participant has had time to review the study information provided, consent will be sought. Informed consent (see Additional File 2), where possible, will be collected in person by a member of the research team prior to the collection of baseline data. All participants will be assigned a study identification code linked to their personal information. All trial data will be recorded and stored in a separate password-protected computer within the Department of Adolescent Medicine at The Children’s Hospital at Westmead and accessible only by the primary research investigators (JG, KS) and any research staff associated with the study. Signed consent forms, questionnaires, and other paper data including the master list linking the personal information of participants and personal ID numbers will be secured in a separate locked cabinet in the Department of Adolescent Medicine and accessible only by the primary research investigators.

### Additional consent provisions for collection and use of participant data and biological specimens {26b}

This trial does not involve collection of biological specimens for storage.

The Australian National Health and Medical Research Council (NHMRC) statement identifies that young people can consent to participate in research without additional consent from parent(s) and/or guardian(s) where the young person has developed the capacity and maturity to understand and consent to participation which involves no more than low risk and where the research aims to benefit the young person (NHMRC, 2007). Adolescents will be able to provide their own consent to be part of the study for the following reasons.

1. In Australia, adolescents can have their own Medicare card and seek independent health advice from 15 years and have access to their own My Health Record from 14 years of age. At 16 years, adolescents are able to consent to all medical procedures except sterilization or gender reassignment surgery. A “mature minor” aged 14–16 years as defined by NSW laws can independently consent to or refuse medical treatment if they have enough intelligence, competence, and a full understanding of the medical treatment proposed [43]. This is referred to as “Gillick Competence.” The Flinders Program is delivered individually without parents present and is unlikely to be effective if the adolescent is unwilling to be part of the program; therefore, the young person’s individual consent is requested in line with Gillick competence guidelines.
2. The program is about adolescents moving towards autonomy in their illness management and being able to consent, rather than just assent, is an important signal about emerging autonomy.
3. If the young person is interested in the research program but the parents may not be interested due to the impact on work commitments, or are time poor, we can deliver the program digitally and thus at no cost or inconvenience to the parent.
4. Some young people are already attending medical care independently often with longstanding relationships with their primary care team.
5. The self-management program is in no way involved in treatment change or scheduling. It works by supporting the young person to manage the agreed treatment plan as determined by their treating team.

Ethics approval has been granted for young people aged 15–18 years to provide their own consent rather than parent/guardian consent for the reasons listed above. Each participant will be asked to sign a consent form to participate in the study. It is important for the study that the young person has consented to working with the health professional and developing increased capacity to make independent decisions as they transition to adult services. The young person’s capacity to consent will be judged with advice from the treating teams who have had a longstanding therapeutic relationship with the young person. In addition, Gillick Competence will be determined by the research team who have extensive experience and expertise in working with adolescents and will also be able to assess the young person’s competency and capacity to consent.

## Interventions

### Explanation for the choice of comparators {6b}

For this study, a randomized controlled trial will be used to compare a modified version of the Flinders Program with usual care for adolescents with a chronic illness. The aim of the intervention is that it will lead to improved therapy adherence beyond that expected in the context of usual care. Study participants will be randomly allocated to one of the following study arms (Fig. 1). The control group will receive standard care which consists of usual patient care practices which are offered by the patient’s individual specialist treating team. In practice, this will be general advice on self-management behaviors to a variable degree and frequency. Participants in the control group will be provided with a general information brochure after randomization providing encouragement for participants to think about the concept and goals of self-management. Initial control participants will be advised that they will be contacted to start the program in 6 months’ time.

Disease-specific measures can identify the differential effects of specific aspects of a disease or its treatment allowing for comparisons [6]. The controls must be on a specified medication and/or treatment for their chronic disease to allow for comparison using both objective (specific markers of illness control) and subjective measures (participant questionnaires). We believe this approach is the most robust measure of optimal control and is the most satisfactory marker available. Specific targeted interventions to promote self-efficacy and develop self-management skills in adolescents with a chronic illness who are transitioning to adult services are not currently routinely delivered in pediatric settings. There are no other self-management programs that are delivered within our institution and the management of therapy adherence is addressed in an ad hoc manner by individual clinicians. This study will also address the lack of evidence available on the effectiveness of self-management programs specifically designed for adolescents with a chronic condition.

### Intervention description {11a}

The Flinders Chronic Condition Self-Management program, commonly referred to as the Flinders Program™ is a validated, internationally recognized program that is designed for adults, and developed from the South Australia HealthPlus Coordinated Care Trials [44] at Flinders University, Adelaide, Australia. It is a structured, generic, individualized program that incorporates the use of specific standardized tools based on the Theory of Planned Behavior (TPB) [45] and utilizing cognitive behavior therapy techniques and motivational interviewing to assess self-management behaviors. The TPB is centered on the assumption that individual’s make logical, reasoned decisions to engage in specific behaviors by evaluating the information which is available to them and that altering specific beliefs can result in positive behavioral changes [45]. The Flinders Program™ involves the application of three key specific assessment tools: the Partners in Health (PIH) Scale, Cue and Response (C&R) Interview, and the Problems & Goals (P&G) Assessment which are founded on the principles of self-management. These assessment tools provide a formal, systematic approach to assessing self-management skills and problems, and support goal setting that leads to the development of a self-management care plan. The care plan incorporates mutually agreed issues and goals which the participant has agreed to work towards over 6–12 months, with monitoring and reviews at 3, 6, and 12 months either via telehealth, in person or phone on a day and time agreed between the young person and health professional [46].

The structured program incorporates the principles of person-centered care which includes a collaborative, consistent, and reproducible approach to the assessment of self-management behaviors, problem solving, goal setting, and individual care planning [46, 47]. This model of self-management has been evaluated and used clinically in a variety of settings, across a range of chronic conditions. Although the Flinders Program™ is generally used in older adult populations, there is some evidence of its use in adolescents with a chronic illness such as cystic fibrosis, asthma, and diabetes [48]. Our approach is unique in that the program is delivered only to the adolescent and does not include standard parental involvement.

The study will be carried out in collaboration with the Flinders Human Behavior and Health Research Unit (FHBHRU) at Flinders University, Adelaide, Australia, in order to inform the development of an adolescent version of the successful and validated Flinders Program™. A modified version of the Flinders Program™ is already being used in the nurse-led self-management support (SMS) clinic in the Department of Adolescent Medicine at The Children’s Hospital at Westmead, Sydney, Australia. The modifications to the Flinders Program™ were piloted in this clinic with collaboration and agreement from the Flinders team. Modifications made to the program were based on our clinical experience and feedback received from the young people. The main modifications included a preference for individual rather than group sessions, a need for flexible goals which are reviewed more frequently than in adults and some minor changes made to the language used in the original Flinders Program™.

The delivery of the intervention is to the individual rather than as a group. A group format may have advantages from the peer support viewpoint, but the researchers have also identified from unpublished pilot work that adolescents will only attend long term if self-management is part of routine health care visits. Contamination at site will be avoided by the multiple specialist clinics in which this intervention is offered. Therefore, it is unlikely that participants in either the intervention or control group will come into contact with each other. The initial session of the structured modified version of the Flinders Program™ is delivered face-to-face in a clinical environment and takes approximately 90 min to complete, in total, with a shorter time frame of 60 min for follow-up sessions which can be delivered via telehealth. This is an important consideration given the current and continuing COVID-19 situation in Australia. We know also from our clinical experience that the initial face-to-face visit appears to improve engagement and trust in the process.

Following randomization, participants in the intervention group will receive a 12-month nurse-led modified version of the Flinders Program™ plus standard care, while participants in the waitlist control group will receive standard care for 6 months before crossing over to the intervention group until all participants are exposed to the program as demonstrated in the study design diagram in Fig. 1. Participants in the intervention group will receive the Flinders Program™ which will be delivered individually, using a combination of face-to-face and telehealth sessions by members of the research team who have been trained in the delivery of the Flinders Program™. The initial face-to-face session will be undertaken in a private room within a clinic environment. Subsequent follow-up sessions will be offered via telehealth modality, either a video conferencing platform or telephone depending on the young person’s preference. Between the delivery of the initial session and 3 months follow-up, participants will receive follow-up consisting of a maximum of 3 phone calls (every 3 weeks), on a date a time agreed between the nurse and the patient. Participants will receive a booster session at 6 months to maintain engagement, monitor progress, and provide feedback and motivation to help them achieve their self-management goals. There is a final follow-up session at 12 months. Figure 1 outlines this process.

#### Initial assessment of self-management

All participants complete the PIH Scale, a self-rated questionnaire, based on their individual perception of their self-management capabilities. The participant rates their answers on a 9-point scale ranging from never (0) to always (8). This is followed by the C&R interview which is administered by a health professional and uses open-ended cue questions to explore in more detail the patients’ responses to the same 12 questions in the PIH scale. This enables the identification of the participant’s understanding and knowledge, as well as the barriers and enablers to self-management. The C&R interview is scored using the same 9-point scale as the PIH scale by a member of the research team trained in the Flinders Program, and the scores are compared and discussed with the participant. Scores of 4 or below (out of a total maximum score of 12) and discrepancies of 3 or more on either side of 4 rated by the participant or the health professional are flagged as issues requiring further discussion and are addressed in the care plan [46]. The health professional uses open-ended questions to identify the participant’s main problems, impact of chronic illness, and feelings to foster collaboration and partnership. This leads to the development of agreed short- to medium-term goals (3 to 6 months) to address the identified problems. These target issues are then integrated into the negotiated individualized care plan which is reviewed at follow-up [46]. The participant is provided with a copy of the care plan which is either sent to their personal email address or posted in the mail directly to them. A clinic letter is sent to the referring specialist and parents/carers indicating that the adolescent has commenced a self-management program which is directed at increasing autonomy and goal setting and does not contain any details of the intervention.

#### Follow-up data collection

- Adherence to prescribed treatment regimen

Baseline observations will be collected from both the waitlist control group and the intervention group before randomization. Potential participants in the intervention group will be contacted via phone by a member of the research team. Follow-up of participants in the intervention group will be undertaken by a member of the research team via phone or telehealth (depending on the young person’s preference) at 3, 6, and 12 months to follow-up on adherence to the prescribed treatment post intervention. Participants who do not respond to 3 attempts to contact them at any time point will be treated as missing data.

Information from the participants will be obtained by completing the PIH scale via a semi-structured interview. Subsequently, participants allocated to the wait list control group will be contacted by phone by a member of the research team and provided with a self-management information brochure, with no further contact from the research team. At 6 months, participants in the waitlist control group will be contacted by phone by a member of the research team and invited to participate in the intervention as described for the initial intervention group.

(b) Markers of illness control and unplanned hospital visits

The research team will obtain standard measures of illness control for all participants who have been recruited into the study and who have consented to clinical information being obtained for research purposes. The control of chronic illness is the best quantitative measure of therapy adherence that is available. This will be undertaken by way of the research team extracting information from the electronic medical records (EMR) and the young person’s primary physician contacted for further information. Data on unplanned hospital visits will be retrieved by the research team from EMR and/or accessed through the young person’s My Health Record if available.

#### Follow-up assessments of self-management

Formal follow-up sessions will be undertaken either via telehealth, in person, or by phone (or email if everything else is not possible) at 3, 6, and 12 months. Face-to-face follow-up sessions, where possible, will be planned to coincide with regular hospital outpatient visits. Telehealth, phone, or email follow-up will be conducted at a mutually agreed time between the healthcare professional and the patient. Measures of illness control, unplanned hospital visits, and questionnaires will be collected at interval periods as indicated in Table 1 using EMR to reduce reporter burden. The focus of follow-up sessions is to review the participant’s progress and provide coaching to achieve their agreed goals and/or set new goals as needed. The health professionals act as a resource person, providing motivation and assisting with problem solving as necessary. Follow-up sessions will follow the same structure as the initial face-to-face session and dependent on the goals of the young person. At 3 months, participants are given a reminder about the booster session which is scheduled at 6 months and their contact details confirmed.

#### Standard care procedures

The control group will receive standard care which consists of usual care practices which are offered by the adolescent’s individual specialist treating team. In practice, this will be general advice on self-management behaviors to a variable degree and frequency. Participants in the control group will be provided with a general information brochure after randomization providing encouragement for participants to think about the concept and goals of self-management. Initial control participants will be advised that they will be contacted to start the program in 6 months’ time. Control participants will be contacted by a member of the research team via phone, 1 month prior to the commencement of the program to advise of the start date which will be coordinated to coincide with the participant’s next clinic visit. The sole purpose of this call is to provide participants with the planned program start date, no intervention work will be provided during this call.

### Criteria for discontinuing or modifying allocated interventions {11b}

The data collected at each assessment point will be used to determine and monitor the safety of the trial participants and to determine if any modifications to the trial protocol are required. A concurrent implementation process will be conducted, and allocated study interventions for trial participants may be modified or discontinued in response to the participant’s request, worsening of disease, or harms. Any changes or modification of the trial protocol will be reflected in the study protocol and the Australian, New Zealand Clinical Trials Registry (ANZCT).

### Strategies to improve adherence to interventions {11c}

Strategies to improve adherence to interventions and maintain engagement are implemented in the study. Participants in the intervention group will receive a maximum of 3 phone calls, spaced 3-weekly and a booster session scheduled at 6 months delivered by a member of the research team. Condition-related data on markers of illness control including laboratory measurements will be obtained from the participants’ EMR.

Participants will also be provided with a confirmation of appointment letter which will be sent via mail or email depending on the participant’s preference prior to each follow-up appointment to maintain adherence to the program, which is consistent with Departmental practice. For participants who have missed a follow-up appointment, a member of the research team will call the participant to reschedule the appointment within a 1-week period if possible. Appointments will be offered via telehealth, phone, or face-to-face consultation. Where possible, face-to-face self-management appointments will be scheduled to coincide with other routine hospital visits to improve attendance, participation, and adherence to the intervention.

### Relevant concomitant care permitted or prohibited during the trial {11d}

Participants should continue to take medications for other comorbid conditions as prescribed by their primary treating team. Both the intervention and control group will continue to receive medical care by their primary treating team throughout the study. Participants enrolled in the “Adolescent Perx Study,” an investigation into the use and applicability of a self-management app in adolescents with chronic conditions conducted within the SCHN, will be excluded to avoid potential contamination of outcomes in both studies.

### Provisions for post-trial care {30}

Post care trial provisions have been outlined in the young person information sheet. In the event of health care needs arising as a direct consequence of trial participation in relation to emotional distress or psychological upset or discomfort, the participant’s primary treatment team and their parents/carers will be notified. Participants are also advised that they may leave the study at any time. Participants ineligible for inclusion in the current study will be offered the self-management clinic as a standard care option if and when required.

### Outcomes {12}

#### Data collection at baseline

Patient demographic data will be collected in person at baseline and will include the following: age, gender, date of birth, diagnosis, year of diagnosis, ethnicity, current medications and therapy prescribed, and email and contact phone number for both the participant and one parent/caregivers. Consent will also be obtained to collect data measures of illness control from EMR. Demographic and baseline characteristics will be summarized using descriptive statistics.

#### Primary outcome measures

Outcome measures and data collection intervals are summarized in Table 2. The primary outcome measure will be a response to treatment as measured by quantitative condition-specific validated markers of improvement in illness control.

#### Measures of illness control

To clarify our primary outcome measure, we provide here a list (not exhaustive) of the more common conditions seen in specialist care in pediatric hospitals which require significant self-management. These include diabetes, other chronic endocrine conditions, asthma, cystic fibrosis, epilepsy, inflammatory bowel disease, haemoglobinopathies, and inherited metabolic disorders [49]. Measures of illness control can be described as a quantitative measurable parameter including laboratory measurement that can be used to identify clinically meaningful endpoints that can be used to assess the status of a known disease. Common features of chronic conditions include prolonged duration, absence of spontaneous resolution, and a lack of curative therapy, requiring ongoing long-term lifestyle modifications and/or pharmacological treatment to be taken consistently and regularly. These characteristics define the chronicity of each of the conditions that will be assessed as part of the study [50]. An improvement of illness control based on clinical experience, given empirical data are lacking, will be assessed by utilizing predetermined measures of illness control, previously developed in conjunction with specialist teams [51] (Table 1). The proportion in each group who achieve improvement in illness control will be described by number and percentage and the difference expressed as relative risk and confidence interval. Measures of effect will be expressed as relative risk with a decrease of 10% from baseline to 12 months. For any conditions not appearing on this list, we will consult with the referring specialist team to determine the appropriate measure of illness control and what would be clinically considered as an improvement of that particular chronic condition. Improvement of illness control will be defined as sustained improvement in measure of disease control over a 12-month period from baseline as defined in Table 1. Illness control will be defined using a binary notation of which a “yes” or “no” answer will be provided, defined as achieving improvement or not. In many conditions, there will be more than one quantitative measure of illness control. For the purposes of outcome analysis, we have selected the most clinically important marker, but will have data on secondary markers of illness control where relevant.

#### Secondary outcome measures

The secondary outcome measures are the number of unplanned or unscheduled health care emergency department visits, quality of life, self-efficacy, time management, health-related distress, and feasibility and acceptability of a modified self-management program designed for adolescents with a chronic condition which include:

- Hospital admissions and emergency department visits due to chronic illness will be obtained from the medical records or My Health Record system if the young person is registered. An unplanned hospital admission is defined as an admission that is not part of routine illness management, not from an admission waiting list and which can be associated with a deterioration of illness control as a result of insufficient self-management practices [51]. These measures will be collected in all study participants at baseline in both standard care and intervention groups as with other measures except the feasibility and acceptability questionnaire at interval periods of 3, 6, and 12 months from baseline. The median and range of numbers of unplanned hospital admissions per person in each group will be described.

#### Partners in Health (PIH) scale

The primary outcome is to determine the effectiveness of using a modified version of the Flinders Program™ as measured by illness control and unscheduled hospital admission as described above. Improvements in self-management will be measured using the validated PIH scale which is incorporated in the Flinders Program™ and provides a formal, low burden scale (usually takes less than 10 min to complete) designed specifically to measure improvements in self-management behaviors and health outcomes over time [46].

The PIH scale assesses self-management capacity using a validated Likert self-rated scale (0 being very little to 8 a lot) for each of the 12 questions covering the principles of self-management including knowledge and treatment of the condition (Q1 and 2); engagement with health care providers (Q3 and 4); access to services (Q5 and 6), monitoring and responding to signs and symptoms (Q7 and 8); physical, social, and emotional impacts of living with a chronic illness (Q9, 10, and 11) and lifestyle risk factors (Q12) [52, 53]. Questionnaires will be completed by all participants independently at commencement of the study as detailed in the study timeline. Scores of less than or equal to 4 are flagged by the health professionals as areas to be addressed. The PIH Scale demonstrates high internal consistency (0.81) and construct validity with a 4-factor analysis for knowledge, symptom management, adherence, and coping accounting for 80% of the variance [54]. The number and percentage of each randomized group who have a score of less than or equal to 4 will be collected at each timepoint 3, 6, and 12 months from baseline.

#### Health-related quality of life

Health-related quality of life will be measured using the Paediatric Quality of Life Inventory (PedsQL) version 4.0, an instrument that measures health-related quality of life (HRQOL) in children and adolescents from the age of 2–18 years. The PedsQL 4.0 Generic Core Scale is designed to incorporate four scales; physical (8 items), emotional (5 items), social (5 items), and school functioning (5 items) as delineated by the World Health Organization [55]. It comprises an adolescent self-report and assesses generic and disease-specific quality of life. For this study, the validated teenage report for ages 13–18 years will be used. This report was chosen as it is brief and easily administered and does not necessitate a large burden on respondents. The teenage report consists of 23 items which participants rate on a 5-point Likert scale with higher scores indicating better quality of life [56, 57]. The psychometric evaluations of the PedsQL 4.0 demonstrated high internal consistency (0.88). Validity was demonstrated using the known-group method, correlations with indicators of morbidity and illness burden [58]. This HRQOL measure was chosen as it is well validated in pediatric patients with acute and chronic conditions and takes into consideration the cognitive developmental needs of young people [55].

#### Time Management Questionnaire (TMQ)

Time management skills have demonstrated a level of effectiveness in both academic and non-academic contexts demonstrated through increased awareness of study habits, reduction in stress, self-regulation of time, and increased levels of self-efficacy [59]. An adaptation of the Time Management Questionnaire (TMQ) developed by Britton & Tesser [60] will be used in this study to assess attitudes and planning of participants. The questionnaire consists of 18 items using a 5-point response scale used consisting of never (1); rarely (2); sometimes (3); often (4); and always (5) with higher scores (except items 2, 3, 9, and 15) correlating to better time management. The TMQ incorporates 3 subscales including short-range planning, time attitudes, and long-range planning. This theoretical model was chosen as it has been validated for use in high school students aged 16–18 years and best suited to our population group [59] and was shown to have a reasonable level of internal consistency across the 3 subscales and total overall scale (0.87) [61]. It is unlikely that participants in our study will not be enrolled in any form of education. In the event that this is the case, we will ask them to omit questions which are specifically related to school.

#### Kessler Psychological Distress Scale (K10)

The Kessler Psychological Distress Scale (K10) is a well-recognized instrument which has demonstrated content and construct validity for measuring non-specific symptoms of psychological distress including behavioral, emotional, cognitive, and psychophysiological manifestations [62]. Initially developed for the US National Health Interview Survey (NHIS), the 10-item scale (K10) has been extensively used in both clinical and general populations including adolescents, and used in a variety of cultural and sociodemographic backgrounds [63]. The K10 scale measures the frequency of symptoms (nervousness, hopelessness, sadness, worthlessness, and fatigue) experienced in the past month using a 5-point Likert scale ranging from none of the time (1) to all of the time (5), producing a total score range of 10–50 with higher scores correlating to more distress [62]. A study conducted by Sampasa-Kanyinga et al. found that the K10 scale has satisfactory psychometric properties for use as a measure of non-specific psychological distress with high internal consistency (0.84) [64].

Adolescents with a chronic condition are more likely to develop psychiatric and behavioral disorders including depression and low self-esteem than their healthy counterparts. Thus, the K10 scale was chosen as it presents as a useful tool in identifying psychological symptoms in participants, and from our previous clinical experience in working with adolescent populations, the accessibility and simplicity of the K10 scale makes it an ideal instrument to use within this population group. [65]. It is not a diagnostic tool.

The mean and standard deviation of the scores for the outcome measures described above will be described in each group and at each time point, following randomization at baseline, 3 months, and 12 months.

#### Feasibility and acceptability Questionnaire (FAQ)

At the end of the self-management intervention participants will be asked to complete a semi-structured qualitative feasibility and acceptability questionnaire (see Additional File 3). This questionnaire, specifically developed for this study by the research team, consists of 5 questions made up of fixed choice responses and free text to provide participants with an opportunity to expand on their responses. The questions relate to the feasibility of the intervention for adolescents, the perceived benefits of participating in the program and their level of satisfaction. If shown to be successful in this RCT, the information obtained from this questionnaire, together with the findings from our systematic review, will be used to improve the self-management program delivery and implemented in the future design and development of the revised adolescent-friendly version of the self-management program.

### Participant timeline {13}

A schematic diagram for participant timeline is shown in Table 2.

### Sample size {14}

The primary outcome of a response to treatment is defined by improvement in disease markers listed in Table 1. We estimate that fewer than 15% of participants would show improvement in disease under usual care, and the intervention would only be considered worthwhile implementing if it can improve responses by a substantial margin. A study conducted by Rhee and colleagues, which evaluated the effectiveness of a self-management program for adolescents with asthma, found overall the intervention group reporting greater improvements in outcome measures; knowledge, self-management skills self-efficacy, and Quality of Life (QOL), than the control group [66]. A study of 54 participants (27 per group) will have 80% power at 5% two-sided alpha to detect a difference in response rates between treatment arms if the rate under usual care is 15% or less and the true difference in proportions is at least 35%, using a chi-square test. To allow for some loss to follow-up, we plan to recruit a total of 60 participants. Participants who withdraw from the study will not be replaced as the power analysis allows for attrition. Participants who withdraw from the study, may have their clinical outcomes determined from their medical records to ensure that the results of the research study can be measured accurately.

### Recruitment {15}

**Fig. 2:**
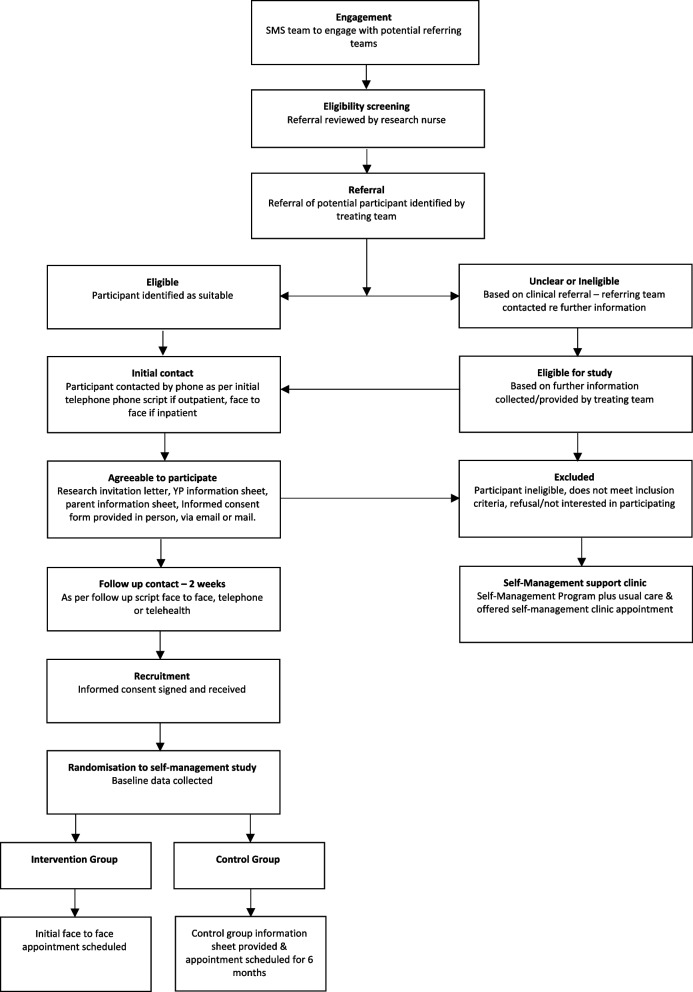
Flowchart for participant recruitment

Potentially eligible participants will be identified by their primary treating teams, who will provide participants with information about the study, prior to their enrollment to determine the young person’s interest and willingness to participate and seek permission for a member of the research team to make contact with them. If permission is granted by the participant, a member of the research team will contact the participant either via telehealth modality, phone contact, or face to face to extend an invitation to participate in the study and provide further information as part of the formal consent process. In addition, all adolescents referred to either the Self-Management Support Service (SMS) in the Department of Adolescent Medicine or the Trapeze Service across both sites of the SCHN (Westmead and Randwick) and who meet the study criteria will be invited to participate in the study by a health professional who is not part of their clinical treatment team (Fig. 2 outlines this process).

Recruitment is planned to be undertaken over an estimated duration of 12 months and will continue until the required number of participants to meet the sample size and power requirements of the study has been reached.

Study information will be advertised on the SCHN staff intranet, and an email sent targeting potential specialist referring teams across the SCHN to assist in referral of eligible young people to the study. Research information flyers will also be used to advertise the study and displayed within various locations, including Adolescent Medicine, outpatient clinics, and adolescent ambulatory care areas at both hospital campuses. In addition, the research team has been promoting the study by collaborating with the primary treating teams to identify eligible patients to increase participant enrollment and meet the target sample size.

## Assignment of interventions: allocation

### Sequence generation {16a}

Following formal consent, the participants will be randomized. Successively, baseline data collection and measures of illness control will be collected for both the control and intervention groups. Randomization will be stratified by disease condition (diabetes, CF, renal, inflammatory bowel disease, other) and will use permuted blocks to maintain balance.

### Concealment mechanism {16b}

Assignment envelopes will be opaque, sealed, sequentially numbered, and opened in the correct order when the intervention is assigned. In accordance with the CONSORT statement (item 10), the envelopes will be prepared and opened by a clinician who is separate from the recruitment of participants [67].

### Implementation {16c}

The random sequence will be prepared by a statistician with no involvement in the trial using computer-generated random numbers. Participants will be sequentially randomized to one of two groups: the intervention group (Flinders Program™) or the control group (standard care for 6 months) before crossing over until all participants have been exposed to the intervention by the end of the trial. Additionally, data will be collected at each step.

## Assignment of interventions: blinding

### Who will be blinded {17a}

This trial is not blinded. As with all studies of this type of intervention, it is not possible to blind trial participants or the researchers who must know what group the participant is assigned to. To avoid potential bias during the statistical analysis of the trial, the data analyst will be blinded until the entire analysis has been completed.

### Procedure for unblinding if needed {17b}

Not applicable to this study.

## Data collection and management

### Plans for assessment and collection of outcomes {18a}

Patient demographic data will be collected at baseline (see Additional file 3). All data collection questionnaires are programmed in Research Electronic Data Capture (REDCap) and include Partners in Health (PIH) Scale, collected at baseline, 3, 6, and 12 months follow-up. Paediatric Quality of Life Inventory (PedsQL) version 4.0 (Teenage Report); Time Management Questionnaire; Kessler Psychological Distress Scale (K10) collected at baseline, 3 months, and 12 months follow-up. Additional data on makers of illness control and unplanned hospital admissions and emergency department visits will be obtained from the EMR at baseline, 3, 6, and 12 months follow-up. Data questionnaire on feasibility and acceptability will be collected on completion of the study at 12 months (see Additional file 4). Data will be collected and stored in REDCap a secure web-based application used for designing and managing clinical research databases. REDCap is an online data capture tool which stores data in a secure and encrypted manner on the University of Sydney server to which we have access [68]. This research data management platform is a secure web-based software platform used for the data management procedures of this trial including data collection, follow-up, and analyses. The built-in component of REDCap has been pre-prepared to accept only variables within given permissible ranges that are consistent with previous entries before going on to the next section to minimize missing values. Data collection forms pertaining to the study will also be stored in REDCap.

Prior to the commencement of the study, research staff completed the Flinders training workshop conducted over 2 days, by the lead research nurse (JG) who is an Accredited Flinders Trainer. All research staff were assessed as competent against current Flinders competency standards. The training was guided by validated manuals (Accredited Trainer: Flinders Chronic Condition Management Program Manual; The Flinders Chronic Condition Program Participant Manual) with electronic copies of all training resources and PowerPoint presentations available to ensure reproducibility and fidelity of the intervention program.

In addition to the delivery of training required for the implementation of the intervention, a self-report evaluation checklist was developed by the research team, specifically for the study, to guide each interview and to assess fidelity ensuring that all key points are covered during each consultation. The self-report checklist is completed immediately after the consultation by the health professional delivering the intervention and is emailed to the research nurse coordinator. Follow-up quality assurance checks will also be completed by the research team as part of the study after each consultation. Information around attendance, aspects of the program covered in the first and subsequent sessions, length of consultation visits, records of all face-to-face consultations and phone contact, and the development of a care plan will be recorded. The training and self-report evaluation checklist serve to minimize technical deviations from the protocol and provide reproducible results.

### Plans to promote participant retention and complete follow-up {18b}

Participants can withdraw from the study at any time. If the patient decides to withdraw from the study, data collected will be retained as outlined in the consent and the reasons for the withdrawal, if provided, will be recorded for subsequent analysis in the interpretation of results. Participants who withdraw from the study will not be replaced as the power analysis allows for attrition.

In the first 3 months, participants will receive a maximum of 3 phone calls from a member of the research team. In addition, an appointment letter is confirming the details of the next appointment is sent to the participant either electronically or sent in the mail which serves as a reminder of the next scheduled appointment to improve adherence and participation to the intervention.

### Data management {19}

Trial data will be recorded and stored in a separate password-protected computer on a secure server within the Department of Adolescent Medicine at The Children’s Hospital at Westmead and accessed only by the primary research investigators (JG, KS) and essential research staff associated with the study. Signed consent forms and other paper data including the master list linking the personal information of participants and personal ID numbers will be secured in a separate locked cabinet in the Department of Adolescent Medicine. The master list will be securely stored separately from other documents containing participant data.

All participant information collected as part of the study will be de-identified on receipt by the investigators and will be coded with participant numbers to ensure confidentiality and anonymity. Digital data collected will be stored on REDCap as described previously.

De-identified digital data will be on a password-protected database on a restricted access network drive. Only the principal investigators will have access to the participant ID number-name reference master list, and this will remain as a separated hard copy and stored separately. The lead research nurse (JG) will randomly double-check a small proportion (10 percent) of the entered data for any outliers and for accuracy.

### Confidentiality {27}

To ensure participant confidentiality, on referral to the study participants from each site involved will be allocated a unique identification (ID) number which will be used for all subsequent data collection, and hard copy and electronic copy stored as per local privacy standards. Any personal information collected from participants will be de-identified using ID numbers and re-identifiable if needed using the master list only. All stored data will be destroyed after 15 years of study completion in accordance with current Health Research Ethics Committee (HREC) requirements, using deletion and shredding protocols.

### Plans for collection, laboratory evaluation, and storage of biological specimens for genetic or molecular analysis in this trial/future use {33}

Not applicable to this study, no samples collected.

## Statistical methods

### Statistical methods for primary and secondary outcomes {20a}

Participant demographic, clinical characteristics, and study outcomes will be presented using standard descriptive statistics, including mean, standard deviation and range or median, quartiles and range for continuous variables, frequencies and percentages for categorical variables, and the Kaplan–Meier method for time to event variables. All statistical analyses will use the intention to treat principle whereby participants are analyzed in their randomized groups, regardless of treatment received. The primary outcome of the number of responses in each treatment arm will be compared using a chi-square test and the difference in proportions presented with a 95% confidence interval. Other study outcomes will be compared between randomized groups using standard tests, including chi-square tests for categorical outcomes, two-sample *t*-tests or nonparametric equivalent for continuous outcomes, Poisson regression models for count data, and log-rank test for time-to-event data. For longitudinal outcomes, statistical models that account for the correlation between repeated observations in the same participant will be used. A more detailed pre-specified statistical analysis plan is forthcoming with completion of the trial and will be made publicly available.

### Interim analyses {21b}

There is no planned interim analysis for this trial.

### Methods for additional analyses (e.g., subgroup analyses) {20b}

Not applicable to this study. No additional subgroup analyses have been planned.

### Methods in analysis to handle protocol non-adherence and any statistical methods to handle missing data {20c}

The primary aim of this study is to evaluate the effectiveness of a modified adolescent-friendly version of an adult self-management program within a clinical environment. To allow for some loss to follow-up, we plan to recruit a total of 60 participants. All available data will be analyzed on an intention-to-treat basis, whereby participants are analyzed according to their randomized treatment allocation group, regardless of whether they actually received the intervention, their subsequent withdrawal or deviation from the intervention. There is no intention to impute missing data.

Participants can withdraw from the study at any time. If the patient decides to withdraw from the study, data collected will be retained as outlined in the consent and the reasons for the withdrawal will be recorded for subsequent analysis in the interpretation of results. Participants who withdraw from the study will not be replaced unless attrition is higher than anticipated as we require 30 participants in each group. Participants who withdraw from the study may have their clinical outcomes determined from their medical records to ensure that the results of the research study can be measured accurately.

### Plans to give access to the full protocol, participant-level data, and statistical code {31c}

The full protocol is planned to be published, as are all group data. Participant-level data can be provided to individuals at the end of the trial period. The trial data release to another research group would be considered on individual request to the Primary Investigators.

## Oversight and monitoring

### Composition of the coordinating center and trial steering committee {5d}

The investigator (KS) who conceived the concept and overall design of the study will be responsible for the oversight of the study and will be in charge of supervising the research team, monitoring the overall coordination, progress, and any modifications to the study protocol as required.

### Composition of the data monitoring committee, its role and reporting structure {21a}

It is anticipated that adverse events or complications will be unlikely from the intervention described in this study. The intervention is about behavior change and does not involve change in medical therapy. As with any research involving adolescents and their families, it is possible that there may be psychosocial and/or physical distress or medical issues that arise in the course of the intervention which are considered low risk and which would be referred back to their treating team. As such, no data monitoring committee will be constituted.

### Adverse event reporting and harms {22}

This project will be conducted by clinical staff from SCHN and who all have experience in working with adolescents. In any clinical research, there is always the chance of psychosocial distress being uncovered. We anticipate that the risk of psychological and/or physical distress due to the intervention being considered low.

When working with adolescents, dealing with anxiety and mood issues are common. In the event that the young person experiences psychological distress or becomes upset during the study, their mood will be assessed, and the young person’s primary clinician and their parent/guardian notified. The young person will be referred to their treating team for management.

There is a risk that remaining on the waitlist control group the young person may experience a short-term deterioration in health as a result of continued non-adherence. This is considered a temporary impact given that the young person would continue to receive usual care provided by their primary treating team and an information brochure on self-management support. With non-adherence to therapy in a very small number of cases, there is a potential for mortality (e.g., diabetic keto-acidosis, coma, or fitting) which can happen even in those adolescents whose chronic conditions are generally well controlled. In the vast majority, non-adherence is non-fatal.

The research team will be responsible for reporting any adverse events which could possibly be attributed to the intervention to the Human Research Ethics Committee. The research team will also notify the patient’s treating team about any events related to chronic condition management or otherwise that are not directly related to the self-management intervention, but which require appropriate follow-up. These events might include medication adverse effects, new symptoms related to physical or mental wellbeing and requests for medication dosage supply or adjustment.

### Frequency and plans for auditing trial conduct {23}

Auditing will be conducted by the primary research nurse coordinator (JG), periodically throughout the study using REDcap’s auditing trial feature. The processes reviewed will include procedures such as, participant eligibility, enrolment, consent procedures, randomization, and reporting of adverse events.

In addition, a self-report checklist is completed by the health professional delivering the intervention and completed immediately following the consultation to ensure consistency of the information provided. Quality assurance checks pertaining to the attendance, aspects of the program covered in the initial and follow-up sessions, length of consultation, record of the number of face-to-face consultations, and phone contact for each participant will also be recorded for auditing trial conduct.

### Plans for communicating important protocol amendments to relevant parties (e.g., trial participants, ethical committees) {25}

We do not anticipate that there will be any significant modifications to the study protocol. Any changes to the research protocol will be submitted to Sydney Children’s Hospital Network Human Research Ethics Committee, The Children’s Hospital at Westmead Sydney, Australia, for approval. Any changes arising will also be resubmitted to the Australian and New Zealand Clinical Trials Registry (ANZCTR) for review.

### Dissemination plans {31a}

On completion of the study, planned dissemination of the results will involve national conference presentations and publication in peer-reviewed journals. Participants have a right to receive feedback about the overall results of the study. The results of the study may be published, and participants can nominate to receive feedback on the consent form. This feedback can be obtained after the study has finished and will be provided in the form of infographics summarizing the key outcomes of the study which will be posted or emailed to the participant’s nominated contact address.

## Discussion

For many young people with a chronic condition, adolescence is a time that may also coincide with psychosocial changes leading to challenging adherence to therapy issues. The normal psychosocial and developmental changes as well as the development of independent life skills common to all adolescents interact with the challenges of living with a chronic condition, in particular, during the transition to adult care, leading to difficulty with both adherence and the attainment of disease self-management skills. In Australia, the common age of transfer from a child centered to adult orientated health care system, typically occurs when the young person is 17–18 years of age or on completion of their final year of secondary school. Therefore, adolescents with a chronic condition often require support to develop self-management health behaviors including adherence to prescribed therapy, health-directed behaviors involving modifications to activity levels and nutrition, and preventative therapies. Despite the evidence in adults which have shown that effective self-management interventions and time management skills have the potential to reduce morbidity and improve health outcomes, there is a lack of evidence-based self-management support models for adolescents. This highlights the significant need for well-established self-management interventions prior to adulthood in order to reduce illness exacerbations and adverse health outcomes of adolescents with a chronic illness. This study has been designed to provide evidence of the effectiveness of self-management programs for adolescents and will be used to demonstrate a better understanding of what is necessary to better engage young people in the self-management process and to inform the development of an adolescent version of the validated Flinders Program™.

## Trial status

Protocol version 3 dated 22nd of April 2021. Recruitment of participants commenced in December 2021 and is anticipated to be completed by October 2023.

## Supplementary Information

**Additional file 1: Supplementary file 1.** Standard protocol Items: Recommendations for Interventional Trials (SPIRIT) Checklist.

**Additional file 2: Supplementary file 2.** Young person consent form.

**Additional file 3: Supplementary file 3.** Feasibility and acceptability questionnaire.

**Additional file 4: Supplementary file 4.** Ethical approval document. 

**Additional file 5: Supplementary file 5.** Trial registration data.

## Data Availability

Access to the final trial dataset will be considered on a case-by-case basis at the discretion of the primary sponsor. Researchers with approval and governance from a Human Research Ethics Committee (HREC) and who provide a methodologically sound proposal will be considered. All data generated or analyzed during this study are included in this published article [and its supplementary information files].

## Abbreviations

CCM: Chronic condition management
C&R: Cue and Response Interview
EMR: Electronic medical records
ANZCTR: Australian New Zealand Clinical Trials Registry
HREC: Human Research Ethics Committee
NHMRC: National Health and Medical Research Council
P&G: Problem and Goals Assessment
PIH: Partners in Health Scale
RCT: Randomized control trial
REDCap: Research Electronic Data Capture
SMS: Self-management support
TPB: Theory of Planned Behavior
NCD: Non-communicable disease
PIS: Patient Information Sheet
PedsQL: Paediatric Quality of Life Inventory
HRQOL: Health-related quality of life
TMQ: Time Management Questionnaire
K10: Kessler Psychological Distress Scale
FAQ: Feasibility and Acceptability Questionnaire
