# Supplementary material for: A randomized controlled trial evaluating the effectiveness of a self-management program for adolescents with a chronic condition: a study protocol
Source: Trials. 2022 Oct 5;23:850. doi: 10.1186/s13063-022-06740-9 (PMC9532816; doi:10.1186/s13063-022-06740-9)
Supplement: Supplementary file 2 — Additional file 2: Supplementary file 2. Young person consent form. [file 13063_2022_6740_MOESM2_ESM.pdf]

Young Person Consent Form

**Study Title**	Effectiveness of a self-management program for adolescents with a chronic illness
**Principal Investigator/s**	Jaunna Gauci, Department of Adolescent Medicine, The Children's Hospital at Westmead. (p: 9845 2446 e: jane.gauci@health.nsw.gov.au)<br>Prof Katharine Steinbeck, Academic Department of Adolescent Medicine, The Children's Hospital at Westmead. (p: 9845 1925 e: Kate.steinbeck@health.nsw.gov.au<br>Prof Sharon Lawn, Flinders Human Behaviour and Health Research Unit, College of Medicine and Public Health, Flinders University, Adelaide. (e: sharon.lawn@flinders.edu.au)<br>A/Prof Susan Towns, Department of Adolescent Medicine, The Children's Hospital at Westmead. (p: 9845 2446 e: susan.towns@health.nsw.gov.au<br>A/Prof Jacqueline Bloomfield, Susan Wakil School of Nursing and Midwifery, Faculty of Medicine and Health, The University of Sydney. (p: 9351 0936 e: jacqueline.bloomfield@sydney.edu.au<br>Dr Jane Ho, Trapeze, The Sydney Children's Hospital Network. (p: 9832 5446 e: jane.ho@health.nsw.gov.au
**Main Study Contact Person**	Mrs Jane Gauci, Department of Adolescent Medicine, The Children's Hospital at Westmead. Phone: 9845 2446 Email: jane.gauci@health.nsw.gov.au

Declaration by Young Person

☐ I ____ have read and understand the Young Person Information Sheet and give my consent to participate in this research study, which has been explained to me in language that I understand or someone has read it to me in a language that I understand by ____

☐ I understand the purposes, procedures and risks of the research study described in the Young Person Information Sheet.

☐ I have had an opportunity to ask questions and I am satisfied with the answers I have received.

☐ I freely agree to participate in this research study as described and understand that I am free to withdraw at any time during the study without affecting my future treatment and The Children's Hospital at Westmead or in the Self-Management Support Clinic.

☐ I understand that I will be given a signed copy of this document to keep.

☐ I give permission for my treating doctor, other health professionals, hospitals or laboratories outside this hospital to release information to The Children's Hospital at Westmead concerning my condition and treatment for the purposes of this research study. I understand that such information will remain private and confidential.

☐ I understand that if I withdraw my from the study, that the research team will not collect any more information about me. I understand that information already collected by the research team about me will be kept and used to ensure that the results of the research project can be measured properly.

☐ I wish to receive a lay summary of the study findings via the following email / post address:

____

NAME OF PARTICIPANT (please print): ____

SIGNATURE OF PARTICIPANT ______________________________________   Date: ____________________

Under certain circumstances (see Note for Guidance on Good Clinical Practice CPMP/ICH/135/95 at 4.8.9) a witness to informed consent is required.*

NAME OF WITNESS* (please print): ____

SIGNATURE OF WITNESS: ____   Date: _______________________

* The Witness is not to be the investigator, a member of the study team or their delegate. In the event that an interpreter is used, the interpreter may not act as a witness to the consent process. Witnesses must be over 18 years of age
