# Supplementary material for: A randomized controlled trial evaluating the effectiveness of a self-management program for adolescents with a chronic condition: a study protocol
Source: Trials. 2022 Oct 5;23:850. doi: 10.1186/s13063-022-06740-9 (PMC9532816; doi:10.1186/s13063-022-06740-9)

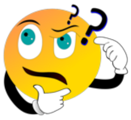

Feasibility and Acceptability Questionnaire (3 & 12 months)

Tell us how helpful the self-management program was to YOU

Date:

i. **What bits of the program did you understand?** (tick all that apply to you)

☐ Setting goals

☐ Working together with a health professional who understands

☐ Problem solving

☐ Developing an action plan

☐ Learning new way to remember taking my medications

☐ Other (please tell us more)

ii. **What didn't you like about the program?** (Tick all that apply to you)

☐ Goal setting

☐ Working together with a health professional

☐ Problem solving

☐ Developing an action plan

☐ Length of the program

☐ Other (please tell us more)

iii. **How happy were you with the program overall**

☐ Very happy ☐ Happy ☐ Neither ☐ Unhappy ☐ Very unhappy

iv. **Would you tell others with a chronic condition about the program?**

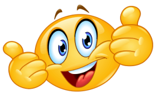 ☐

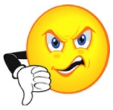 ☐

If no, why not?

v. **Can you think of any ways we can make the program better?**

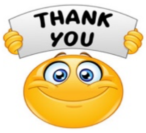

Supplement: Supplementary file 3 — Additional file 3: Supplementary file 3. Feasibility and acceptability questionnaire. [file 13063_2022_6740_MOESM3_ESM.pdf]
