# Supplementary material for: A randomized controlled trial evaluating the effectiveness of a self-management program for adolescents with a chronic condition: a study protocol
Source: Trials. 2022 Oct 5;23:850. doi: 10.1186/s13063-022-06740-9 (PMC9532816; doi:10.1186/s13063-022-06740-9)
Supplement: Supplementary file 4 — Additional file 4: Supplementary file 4. Ethical approval document. [file 13063_2022_6740_MOESM4_ESM.pdf]

The Sydney children's Hospitals Network

care, advocacy, research, education

Contact for this correspondence:
Research Ethics Office
Research Ethics Support Officer
Phone: (02) 9845 1253
Facsimile: (02) 9845 1317
Email: SCHN-ethics@health.nsw.gov.au

Corner Hawkesbury Road
and Hainsworth Street
Locked Bag 4001
Westmead NSW 2145
Sydney Australia
DX 8213 Parramatta
Tel +61 2 9845 0000
Fax +61 2 9845 3489
http://www.schn.health.nsw.gov.au/
ABN 53 188 579 090

9 February 2021

Mrs Jaunna Gauci
Department of Adolescent Medicine
The Children's Hospital at Westmead

Dear Mrs Gauci,

**HREC Reference:**	**2020/ETH02766**
**Project title:**	**A randomised controlled trial evaluating the effectiveness of a self-management program for adolescents with a chronic illness**
**Sites:**	**The Children's Hospital at Westmead**<br>**Sydney Children's Hospital, Randwick**

Thank you for submitting the above project for single ethical and scientific review. This project was first considered by the Sydney Children's Hospitals Network Human Research Ethics Committee ("the Committee") at its meeting **20 November 2020** and subsequently by the Executive of SCHN HREC on the **8 February 2021**.

The HREC has been accredited by the NSW Department of Health as a lead HREC under the model for single ethical and scientific review, and by the National Health and Medical Research Council as a certified committee in the review of multi-centre clinical research projects.

This HREC is constituted and operates in accordance with the National Health and Medical Research Council's *National Statement on Ethical Conduct in Human Research* and *CPMP/ICH Note for Guidance on Good Clinical Practice.*

I am pleased to advise that the Committee has granted ethical approval of this research project. Your approval is valid for five (5) years, effective the date of this letter.

This application has been assessed in accordance with, and meets the requirements of the National Statement on Ethical Conduct in Human Research (2007).

The documents reviewed and approved by the Committee are:

Document	*Version*	*Date*
Parent Guardian PIS	V1	Aug 2020

Document	*Version*	*Date*
Young Person Information Sheet	V1	Aug 2020
Young person Consent	V1	Aug 2020
Research Information Flyer	V1	Aug 2020
Quantitative data collection sheet	V1	Aug 2020
Time Management questionnaire	V1	Aug 2020
Initial research phone script	V1	Aug 2020
Booster Session phone script	V1	Aug 2020
Follow-up research phone script	V1	Aug 2020
Invitation Letter	V1	Aug 2020
Control group general Information Sheet	V1	Aug 2020
Demographic Checklist	V1	Aug 2020
Feasibility and Acceptability Questionnaire	V1	Aug 2020
Health Professional Self-report Checklist	V1	Aug 2020
Cue and Response interview	-	2020
Kessler 10 and scoring	-	2020
Modified Care Plan	V1	Aug 2020
PedQL 4.0 Teenager report (ages 13-18)	V4.0	Apr 2004
Partners in Health Scale	-	Mar 2016
REGIS Project Registration	-	Received 06 Nov 2020
HREA	V3	08 Dec 2020
Protocol	V2	30 Jan 2021
Responses Committee Form	-	02 Feb 2021

Please note the following conditions of approval:

1. The Coordinating Investigator will immediately report anything which may warrant review of ethical approval of the project in accordance with the SCHN adverse event reporting policy.

2. All proposed changes to the research protocol, including the conduct of the research, changes to site or personnel, or an extension to HREC approval, are to be provided to the HREC or its delegate for review before those changes can take effect.

3. The HREC will be notified, giving reasons, if the project is discontinued at a site before the expected date of completion.

4. The co-ordinating investigator will provide an annual report to the HREC on the anniversary of this approval letter, and a final report on completion of the study.

5. Your approval is valid for five (5) years from the date of the final approval letter. If your project extends beyond that five year period and you are still actively recruiting you will be required to resubmit your application incorporating any amendments within six (6) months of that approval expiry date. If your project is in follow up on, or analysis, please submit and application for amendment to extend the approval period. Ethics approval can be extended for a period of twelve (12) months at a time.

6. In the event of a project **not having commenced** within 12 months of its approval, the approval will lapse and reapplication to the HREC will be required.

Should you have any queries about the HREC's consideration of your project please contact the Research Ethics Support Officer on (02) 9845 1253.

You are reminded that this letter constitutes ethical approval only. You must not commence this research project at a site until separate authorisation from the Chief Executive or delegate of that site has been obtained. A copy of this letter must be forwarded to all site investigators for submission to the relevant Research Governance Officer.

The SCHN HREC wishes you every success in your research.

Yours faithfully

Sarah Garnett

Associate Professor Sarah Garnett
Chair, Sydney Children's Hospitals Network Human Research Ethics Committee
Sydney Children's Hospitals Network Human Research Ethics Committee

NB: All clinical trials must now be registered on a publicly accessible registry such as the Australian New Zealand Clinical Trials Registry. For further information please go to www.anzctr.org.au. **Please provide this office with a copy of your registration number for our records if you have not already done so.**
