# Supplementary material for: A randomized controlled trial evaluating the effectiveness of a self-management program for adolescents with a chronic condition: a study protocol
Source: Trials. 2022 Oct 5;23:850. doi: 10.1186/s13063-022-06740-9 (PMC9532816; doi:10.1186/s13063-022-06740-9)
Supplement: Supplementary file 5 — Additional file 5: Supplementary file 5. Trial registration data. [file 13063_2022_6740_MOESM5_ESM.docx]

Trial registration data

| **Data category** | **Information** |
| --- | --- |
| Primary registry and trial identifying number | Australian and New Zealand Clinical Trials Registry, (ACTRN12621000390886). |
| Date of registration in primary registry | 08 April, 2021 |
| Type of registration | Prospectively registered |
| Source(s) of monetary or material support | The Department of Adolescent Medicine and the Academic Department of Adolescent Medicine at The Children’s Hospital at Westmead, Sydney, NSW, Australia. |
| Primary sponsor | Mrs Jaunna Gauci |
| Secondary sponsor(s) | None |
| Contact for public queries | Mrs Jaunna Gauci [Email: [jane.gauci@health.nsw.gov.au](mailto:jane.gauci@health.nsw.gov.au)] |
| Contact for scientific queries | The Department of Adolescent Medicine, The Children’s Hospital at Westmead, Locked Bag 4001, Westmead NSW 2145. |
| Public title | A randomised controlled trial evaluating the effectiveness of a self-management programme for adolescents with a chronic illness |
| Scientific title | A randomised controlled trial evaluating the effectiveness of a self-management programme for adolescents with a chronic illness |
| Countries of recruitment | Australia |
| Health condition(s) or problem(s) studied | Chronic illness; medication adherence |
| Intervention(s) | Intervention: 12-month nurse led modified version of the Flinders Program plus standard care |
|  | Waitlist control: Standard care for six months before crossing over to the intervention group until all participants are exposed to the intervention |
| Key inclusion and exclusion criteria | Minimum eligible age for study: ≥15years. Sexes eligible: both males and females. Accepts healthy volunteers: no |
|  | Inclusion criteria: adolescents aged between 15-18 years, who have a chronic physical condition needing regular health care and who have been identified by their treating team as having unsatisfactory control of this condition and requiring the development of self-management skills. |
|  | Exclusion criteria: adolescents who lack competence in English, who have an intellectual disability or have a chronic condition with no routinely measurable quantitative condition specific validated marker of illness control that can be used to identify clinically meaningful endpoints. |
| Study type | Interventional |
|  | Allocation: randomised; Intervention model: parallel assignment; Masking: not used; Other design features: waitlist |
|  | Primary purpose: treatment |
|  | Phase: not applicable |
| Date of first enrolment | December 2021 |
| Target sample size | 60 |
| Recruitment status | Recruiting |
| Primary outcome(s) | Unplanned or unscheduled hospital admission and emergency department visits due to chronic illness. |
| Key secondary outcomes | Health related quality of life, change in self-management behaviours over time, time management, health related distress, feasibility and acceptability of the intervention. |
